# Trends in socioeconomic inequalities in life expectancy and lifespan variation in Chile

**DOI:** 10.3389/fpubh.2024.1404410

**Published:** 2024-06-27

**Authors:** Nicolas Silva-Illanes

**Affiliations:** ^1^Sheffield Centre for Health and Related Research, The University of Sheffield, Sheffield, United Kingdom; ^2^Program of Health Policy, Systems, and Management, Institute of Population Health, University of Chile, Santiago, Chile

**Keywords:** life expectancy, health inequalities, mortality, time trends, lifespan variation

## Abstract

**Background:**

Socioeconomic disparities in life expectancy are well-documented in various contexts, including Chile. However, there is a lack of research examining trends in life expectancy inequalities and lifespan variation over time. Addressing these gaps can provide crucial insights into the dynamics of health inequalities.

**Methods:**

This study utilizes data from census records, population surveys, and death certificates to compare the life expectancy and the lifespan variation at age 26 of individuals according to their rank in the distribution of years of education within their own birth cohort. The analysis spans three periods (1991, 2002, and 2017) and focuses on two educational groups: individuals in the first (lowest) quintile and tenth (highest) decile of educational attainment. Changes in life expectancy are disaggregated by major causes of death to elucidate their contributions to overall trends.

**Results:**

Consistent with existing literature, our findings confirm that individuals with lower education levels experience lower life expectancy and higher lifespan variation compared to their more educated counterparts. Notably, by 2017, life expectancy for individuals in the lowest quintile of education has caught up with that of the top decile in 1991, albeit with contrasting trends between genders. Among women, the gap has reduced, while it has increased for males. Moreover, lifespan variation decreased (increased) over time for individuals in the tenth decile (first quintile). The leading causes of death that explain the increase in life expectancy in women and men in the tenth decile as well as women in the first quintile are cardiovascular, cancer, respiratory and digestive diseases. In the case of males in the first quintile, few gains have been made in life expectancy resulting from cancer and a negative contribution is associated with digestive conditions.

**Conclusions:**

This study underscores persistent socioeconomic disparities in life expectancy in Chile, emphasizing the importance of ongoing monitoring of health inequalities across different demographic segments. The gender-specific and educational gradient trends highlight areas for targeted interventions aimed at reducing health disparities and improving overall population health outcomes. Further research is warranted to delve into specific causes of death driving life expectancy differentials and to inform evidence-based policy interventions.

## 1 Introduction

Chile has experienced considerable gains in life expectancy in a short period of time relative to the time other high income countries have taken to achieve the same results (1, 2). It is well known that a negative correlation exists in most countries between socioeconomic position and life expectancy (3).^1^ Chile is not exemption in this sense; there is ample evidence of a socioeconomic gradient in life expectancy.

Fuentes-García et al. (5) researched socioeconomic inequalities in life expectancy among a cohort of older adults in Santiago. Looking at life expectancy at age 60, the study found that, while the poorest group could expect to live a further 16 years, the corresponding figure was 23 years for the richest group.^2^ In a comparative study published by the OECD which analyzed life expectancy at age 25 by educational status in 23 countries, Chile ranked 19th for males and 22nd for females, with a gap of 10.9 and 7.6 years respectively between those with tertiary education and those without tertiary education (3). More recently, Espinoza et al. (6) reported a gap of 7.6 years in life expectancy at age 25–29 between individuals with no education or only pre-school education and those who achieved tertiary education.

Using area-level information in Santiago to compare life expectancy at birth between the first and ninth deciles of subcity units in terms of socioeconomic status, Bilal et al. (7) found a 8.9-year gap for men and a 17.7-year gap for women. Edwards et al. (8) used administrative data of pensioners and found a 3-year gap in life expectancy at age 65 between high and low earners, for both men and women.^3^ Moreno et al. (9) also focus on life expectancy among the older adults. Using information from a population survey linked to vital statistics, the study compared life expectancy at age 60 for individuals with public and private health insurance, finding a gap of 4.9 years for men and 5.6 years for women.

In many countries, socioeconomic inequalities in life expectancy have increased over time in absolute terms. Evidence from the USA and the UK suggests that, as well as witnessing an increasing gap in life expectancy between socioeconomic groups, some groups in society have experienced a stagnation or even reduction in life expectancy in recent years (10–13).^4^

The phenomenon observed in the US and UK of an increasing gap in life expectancy by socioeconomic groups has also been identified in nations with much lower income inequality levels, such as Denmark, Finland and Sweden (14–18). Moreover, comparative analyses suggest that Nordic countries have similar or higher absolute and relative inequalities in age-adjusted mortality and life expectancy compared to other countries on the continent (3, 19, 20).

Nevertheless, in Spain, which has one of the lowest socioeconomic gaps in age-adjusted mortality in Europe (19, 20), absolute inequality in life expectancy between educational groups has also increased (21) over the last 60 years. In summary, notwithstanding their experience of increased life expectancy across the population as a whole, many high income countries have also faced widening socioeconomic inequalities in life expectancy at the same time.

Within mortality research, two main patterns of changes in mortality over time have been identified. A “shift” to the right in the distribution of death (also referred to as “mortality delay”), with little change in the shape of the distribution (22–25), and a “compression” of mortality, with a higher proportion of deaths occurring in a narrower age interval (26, 27). The compression of mortality has been studied using several measures that account for the variability of age at death in a given population, which are usually referred to as “lifespan variation” (28). Furthermore, it has been suggested that lifespan variation should be monitored over time to help to detect patterns in mortality changes (28, 29).

Increasing lifespan variation indicates that mortality at younger ages is not decreasing as fast as at older ages. Moreover, a growth in lifespan variation may indicate an increase in mortality at younger ages. Evidence shows that socioeconomically disadvantaged groups have higher lifespan variation than economically advantaged groups (3, 30, 31). Moreover, lifespan variation has been shown to decrease more among higher socioeconomic groups (18, 21, 32, 33).

This study aims to assess, for the first time, trends in socioeconomic inequalities in life expectancy and lifespan variability in Chile, and to understand the contribution of different diseases to changes in life expectancy in different socioeconomic groups over time.

## 2 Materials and methods

### 2.1 Data

The analysis focuses on educational inequalities in life expectancy at age 26 at three time periods: 1991, 2002, and 2017. For each period, data on population at risk and death counts by age, sex and number of years of education were obtained. This information was collected from two different sources. Data relating to population at risk was retrieved from census micro-data (34). Data for death counts were obtained from the mortality database, which is administered jointly by the National Institute of Statistics, the Ministry of Health and the Civil Registry (35).

From each death record, information about education and leading causes of death was obtained. Information regarding the deceased person's education is provided by the next of kin of the deceased person, while the immediate cause of death is taken from death notification documents, prepared by a medical doctor.^5^ Data quality and plausibility checks for causes of death are implemented by the above-mentioned public bodies. According to the World Health Organization, the quality of information on causes of death in Chile is high^6^ (37). Causes of death were grouped into seven categories, which are among the leading causes of death (38): cancer, cardiovascular, digestive, infectious, mental and behavioral, respiratory and other causes. Details of how diseases were categorized into these groups are reported in the Supplementary material.

Micro-data is available for the censuses of 1992, 2002, and 2017.^7^ Moreover, information relating to years of education in the mortality database is missing for 1992. In order to make up for this, the estimates for the period 1991 were obtained using data of population at risk from the census of 1992 and information about mortality for 1991.

Information on education contained in both the censuses and the mortality databases includes details of the highest level of education attained and number of years of education within the highest level of education achieved. From this, an ordinal variable taking values from 0 to 20—“years education”—was built. The Supplementary material describes the mapping from information on years of education attained in a given educational level to the ordinal variable years of education.

### 2.2 Definition of educational groups

As highlighted in the literature (39–43), the proportion of individuals at each educational level (e.g., primary school, secondary school, etc.) changes across birth cohorts, making educational categories unsuitable as a ranking measure over time. In this context, Chile has experienced a significant increase in educational attainment over the last century (see Supplementary material). For instance, in 2017, an 87-year-old woman with lower secondary education (i.e. 8 years or less) would be approximately in the highest decile of educational attainment within her birth cohort, whereas a 30-year-old woman in 2017 with the same level of education would fall into the lowest decile for her birth cohort.

To address this limitation, we define the educational rank of individuals based on the distribution of years of education (ranging from 0 to 20 years) for each birth cohort, following (39). Each individual's rank was computed in relation to the distribution of education among individuals aged 26-30 years old,^8^ when that individual was 26 years old.^9^ For instance, the rank of someone who was 90 years old in 2017 was defined in relation to the distribution by years of education among individuals aged 26–30 years in 1953.

Distribution by years of education for each year relies on information from censuses (1920, 1930, 1940, 1952, 1960, 1970, 1982, 1992, 2002, and 2017) and a repeated cross-sectional survey (CASEN survey) which is representative of the Chilean population (1990, 1994, 1996, 1998, 2000, 2006, 2009, 2011, and 2015). For years for which no information is available, distribution by years of education was computed using linear interpolation based on the two closer adjacent known values (e.g. the distribution for 2016 was computed based on information for 2015 and 2017—see Supplementary material for more details).

Measurement error regarding education reported by the next of kin of the deceased person is a possibility. Moreover, it is less likely for people to err the educational category attained by their deceased relative (e.g. primary or secondary education) than to be mistaken about the years of education attained within a category (e.g. 5 or 6 years of primary education). Based on this premise, we choose to compare the life expectancy of individuals in the first quintile against those in the tenth decile, as these educational ranks closely match educational categories over time.^10^

Given the educational levels, the distribution of years of schooling is not smooth, and the upper limit (lower limit) of the first quintile (the tenth decile) is unlikely to coincide with the steps in the years-of-schooling distribution. For instance, among women aged 26–30 years in 2017, about 15% attained 12 years of education or less, while about 50% attained 13 years of education or less. Therefore, the first quintile includes those with 12 years, and some, but not all, of those with 13 years. In order to allocate those with 13 years to the first quintile, a composition-adjusted method was used (39, 44–46), where numbers of deaths and the population at risk in the educational categories with individuals belonging to adjacent ranks was proportionally categorized in each rank. In the example above we assume that a random one out of seven people who attained 13 years of education belong to the first quintile.^11^

### 2.3 Estimation

Age-specific mortality rates were estimated for the two educational groups by sex, for each year. Based on this information, four separate period life tables were computed and life expectancy at age 26 (*e*_26_) and the modal age at death (*M*) were estimated for each year. We have chosen life expectancy at age 26, because it can be assumed that most individuals would have completed the majority of their education by that age. Life expectancy was estimated with a maximum age of 100 years, because the mortality database is truncated at that age.

We measure lifespan variability using the life disparity estimator (*e*^†^). Life disparity is the population-average of the remaining life expectancy at the age when death occurs (47):


$$\begin{array}{l} e^{†}=\int_{x=0}^{w}e_{x}f_{x}dx, \end{array}$$

where *w* is the maximum lifespan, and *f*_*x*_ is the life table distribution of deaths, with ∑*f*(*x*) = 1.

As we have obtained estimates for small sub-populations, mortality rates tend to be unstable. As well as estimates based on the observed mortality rates, we therefore produced life tables based on smoothed mortality rates. Raw mortality rate estimates often fluctuate due to the inherent variability in the mortality process within the at-risk population. This variability can cause mortality estimates to deviate from a steadily increasing trend during middle and old age, resulting in implausible patterns (48). Smoothing is particularly crucial for the oldest age groups, where the number of exposures is low and data is sparse. For this purpose we used a parametric model, known as the gamma-Gompertz-Makeham model. Following Missov et al. (49) it was assumed that at each age death counts follow a Poisson distribution:


D(x)~Poisson(E(x)μ(x)),

where *E*(*x*) is the age-specific offset and μ(*x*) is the gamma-Gompertz-Makeham mortality hazard at each age:


(1) $$\begin{array}{l} \mu \left(x\right)=\frac{ae^{bx}}{1+\frac{\alpha \gamma }{b}\left(e^{bx}-1\right)}+c. \end{array}$$

Equation (1) has four parameters. Parameter *a* captures the base level of mortality, *b* is related to the age-specific force of mortality, *c* accounts for an age-independent risk of death from all causes and γ accounts for the effect of frailty. Independent models were fitted for each year, sex and educational group. Models were estimated using maximum likelihood.

The actuarial method proposed by Pollard (50) was used to assess the contribution to the changes in mortality rates by age and leading causes of deaths for each educational group. This method enable us to examine changes in life expectancy over two periods of time with relation to age and causes of death. With *y*1 and *y*2 two calendar years, changes in life expectancy at age 26 between both periods ($e_{26}^{y2}-e_{26}^{y1}$) for a given educational rank can be decomposed as follows:


$$e_{26}^{y2}-e_{26}^{y1}=\frac{1}{2}\sum_{i=1}^{k}\sum_{x=0}^{100}{(}_{i}m_{x}^{y1}{-}_{i}m_{x}^{y2}){(}_{x}p_{26}^{y2}e_{x}^{y1}{+}_{x}p_{26}^{y1}e_{x}^{y2}),$$

where *i* is one out of the seven mutually exclusive causes of death, _*i*_$m_{x}^{y}$ corresponds to the mortality rate resulting from cause *i*, at age *x* during the period *y* and _*x*_$p_{26}^{y}$ accounts for the probability of living from age 26 to age *x* at the period *y*.

## 3 Results

Table 1 shows life expectancy at age 26, life disparity and the modal age at death. For each year, the first row shows the results based on the observed mortality rates, while the data in parentheses represents results obtained from smoothed life tables. Estimates of life expectancy and life disparity differ little between the life tables obtained using observed mortality rates and those built based on smoothed life tables. As it could be expected, modal age at death differs depending on methods as a result of random fluctuation when using the life tables based on observed mortality.

**Table 1:** Summary of life-table statistics.

	**Women**						**Men**					
	**First quintile**			**Tenth decile**			**First quintile**			**Tenth decile**		
	(*e*_26_)	**(e** ^†^ **)**	**(M)**	(*e*_26_)	**(e** ^†^ **)**	**(M)**	(*e*_26_)	**(e** ^†^ **)**	**(M)**	(*e*_26_)	**(e** ^†^ **)**	**(M)**
1991	50.1	10.9	81	57.4	10.8	80	46.0	12.9	76	51.2	11.3	78
	(50.3)	(11.1)	(80)	(57.5)	(10.7)	(80)	(46.3)	(12.8)	(78)	(51.3)	(11.4)	(78)
2002	55.9	10.8	88	60.2	10.4	88	49.9	13.7	83	55.3	11.4	81
	(56.1)	(10.8)	(87)	(60.2)	(10.3)	(90)	(50.1)	(13.7)	(85)	(55.4)	(11.3)	(83)
2017	57.3	11.6	90	61.0	8.7	92	50.4	14.3	87	56.8	9.3	88
	(57.3)	(11.7)	(92)	(60.0)	(8.8)	(90)	(50.7)	(14.2)	(86)	(56.8)	(9.2)	(86)

(*e*_26_), life expectancy at age 26; (e^†^), life disparity; (M), modal age at death.

Data in parentheses represents results obtained from smoothed life tables.

In 2017, the life expectancy of individuals in the lowest socioeconomic quintile matched that of those in the highest socioeconomic decile in 1991. Specifically, a 26-year-old woman in the first quintile could expect to live up to age 83.3, equivalent to the life expectancy of a woman in the tenth decile in 1991, who could expect to live up to age 83.4. Similarly, 26-year-old men in the first quintile in 2017 could expect to live up to age 76.4, slightly below the life expectancy of men in the tenth decile in 1991, who already had a life expectancy of 77.2 years at the same age.

The trends in the socioeconomic gap exhibit distinct patterns for women and men. From 1991 to 2017, the life expectancy increase for women in the lowest socioeconomic quintile was twice that of women in the highest quintile, resulting in a reduction of the gap in life expectancy for both groups, in absolute and relative terms. Conversely, the gap in life expectancy between the lowest and highest quintiles expanded for men over the same period, both in absolute and relative terms. In 1991, the gap stood at 4.4 years, increasing to 5.6 years by 2017.

**Figure 1:**
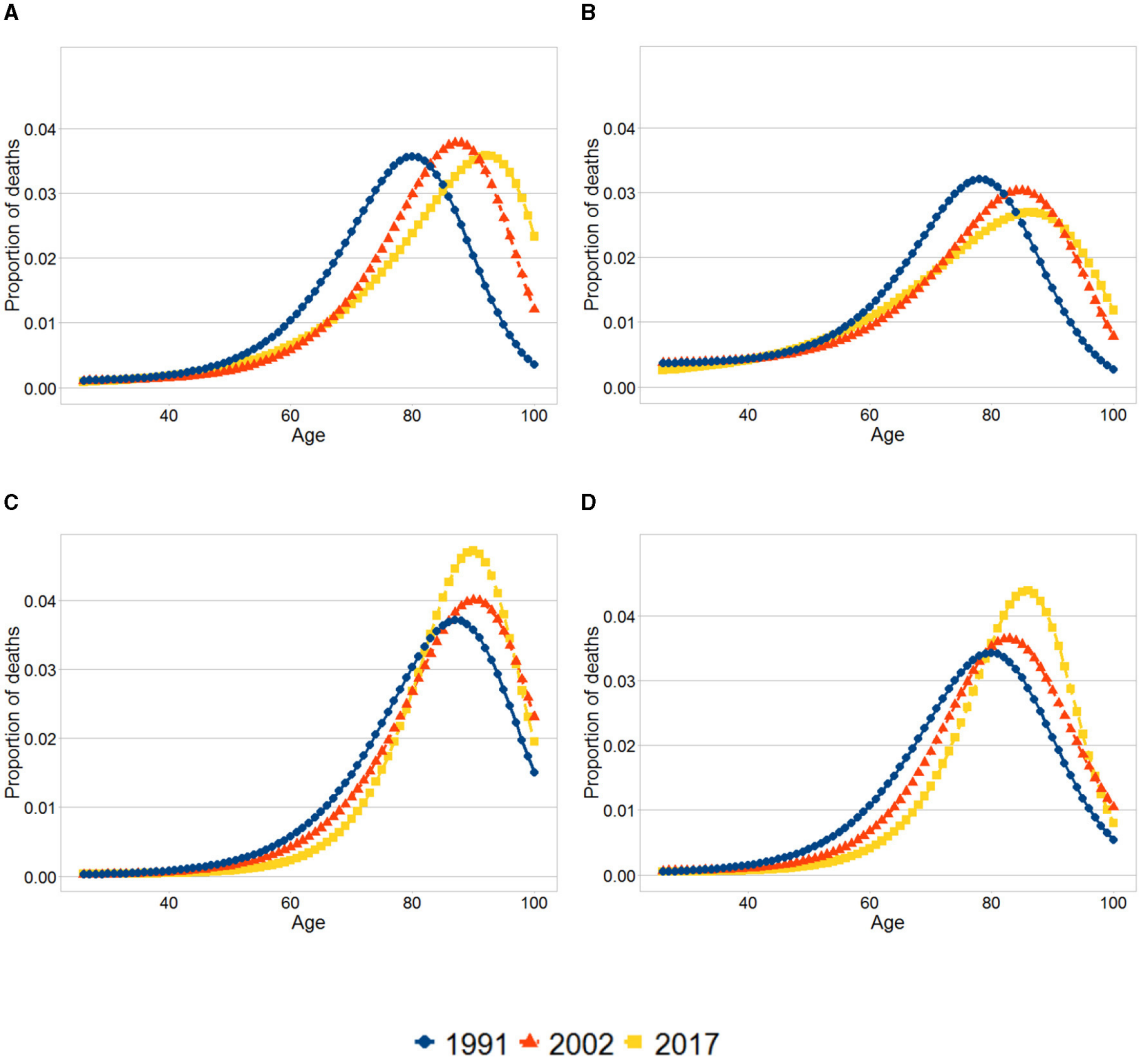
Smoothed age-at-death distributions. **(A)** Women, First quintile. **(B)** Men, First quintile. **(C)** Women, Tenth decile. **(D)** Men, Tenth decile. The graphs show the distribution of deaths at each age for the first quintile and tenth decile for each sex and year. The distributions were obtained using the results from the smoothed life tables.

We now turn to the analysis of lifespan variability. As expected, for both men and women lower life expectancy among individuals in the first quintile is paired with higher life disparity relative to the tenth decile. Moreover, for both sexes, there is little difference in the modal age-at-death between the first quintile and the tenth decile. Accordingly, lower life expectancy and higher life disparity for those in the first quintile is arguably mainly due to a higher proportion of deaths concentrated in younger ages in the first quintile. This can be seen in Figure 1, where for both men and women the modal age at death (the highest point in the curve) at each year is similar between both educational groups, and at each year the mass of the distribution among the first quintile is more concentrated on the left compared to the tenth decile.

For both sexes and both educational groups, evidence exists of a shift in mortality with the modal age at death increasing. The pattern of compression of mortality differs between the first quintile and the tenth decile. For both men and women in the first quintile life disparity went up over time, demonstrating an increase in variability in the distribution of deaths. Furthermore, between 2002 and 2017 a slight increase in the mortality rates takes place for some age groups (around age 50 years) in the first quintile. In contrast, in the tenth decile there is evidence of compression of mortality. These changes are reflected in Figures 1C, D, where there is an increasing proportion of deaths concentrated around the modal age at death in the tenth decile as oppose to the first quintile.

**Figure 2:**
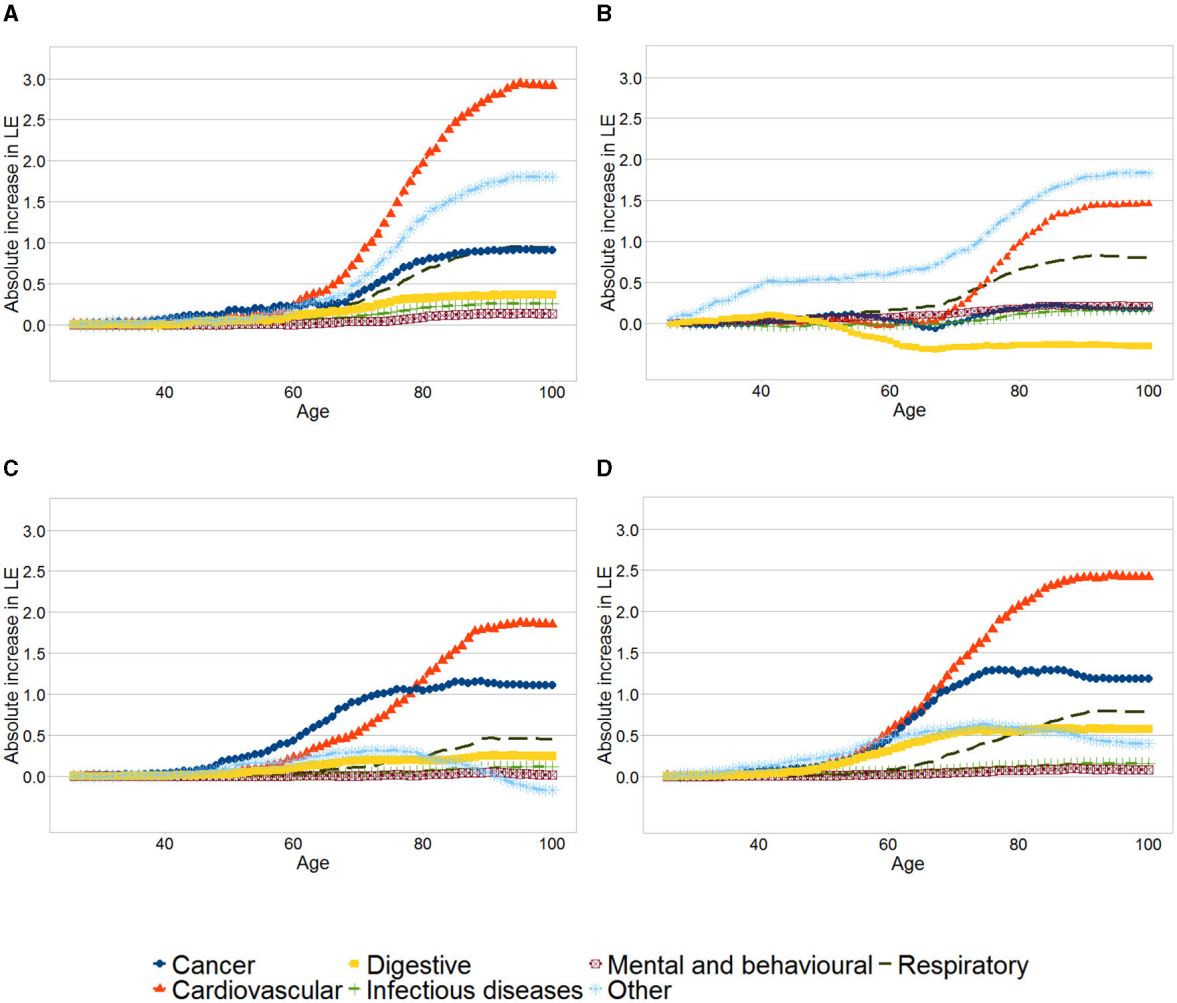
Contributions to changes in mortality rates by age and leading causes for each educational group. **(A)** Women, First quintile. **(B)** Men, First quintile. **(C)** Women, Tenth decile. **(D)** Men, Tenth decile. The graphs show the cumulative increase in life expectancy across age due to groups of diseases, between 1991 and 2017 for the first quintile and tenth decile for each sex and year. The total increase in life expectancy due to each group of diseases corresponds to the quantity shown at age 100.

Figure 2 shows the results of the analysis of contributions to changes in mortality rates by age and leading causes of deaths for each educational group. The graphs show the accumulated increase in life expectancy due to the reduction of deaths at each age, by the leading cause of death. The total increase in life expectancy due to each group of diseases corresponds to the quantity shown at age 100. For example, the increase in life expectancy as a consequence of a reduction of deaths due to cardiovascular diseases between age 26 and 80 for men in the first quintile was 1 year, and the total increase in life expectancy (between age 26 and 100) due to this cause is nearly 1.5 years.

Across the four panels, the reductions of deaths due to cardiovascular diseases, cancer and respiratory diseases are among the leading causes explaining the increase in life expectancy. For both women and men, the gains in life expectancy as a result of other causes were higher among those in the first quintile *vis a vis* those in the tenth decile. Compared to men in the tenth decile, men in the first quintile have experienced a lower increase in life expectancy due to cardiovascular diseases, with roughly no increase below 70 years.

Increased life expectancy due to reduction in cancer mortality was negligible among men in the first quintile, while men in the tenth decile have increased their life expectancy by 1.18 years due to this cause. Furthermore, major differences exist in the contribution of mortality resulting from digestive conditions - life expectancy for men in the first quintile reduced by 0.27 years because of this, compared to an increase of 0.57 years among men in the tenth decile.

As already mentioned, women in the first quintile experienced a higher life expectancy increase than those in the tenth decile. As it is shown in Figure 2A women in the first quintile experienced slightly smaller gains in life expectancy as a result of lower number of cancer deaths and larger gains because of reduction in cardiovascular and respiratory mortality than those in the tenth decile.

## 4 Discussion

The findings of this study shows that in Chile for both sexes there is a gap in life expectancy by education at each point in time. Notably, individuals in the lowest socioeconomic quintile in 2017 have only recently reached the life expectancy levels of those in the highest socioeconomic decile in 1991. Furthermore, we were able to conclude that between 1991 and 2017, while absolute and relative inequality in life expectancy at age 26 by education have decreased over time among women, they have increased for males.

At each year, for both sexes the modal age at death is similar between the first quintile and the tenth decile, with differences in life expectancy explained mostly by higher mortality at younger ages among the less educated. In addition, this paper has shown that, while life disparity has decreased for the tenth decile, it has increased for the first quintile, particularly among males. This calls for close monitoring of patterns of mortality among disadvantaged groups.

Increases in life expectancy as a result of changes in specific causes of death can mainly be attributed to cardiovascular diseases, cancer, respiratory and digestive disease, the main causes of death in Chile (38). There are some similarities and many differences in the pattern of changes between men and women. In both cases there is a bigger increase in life expectancy due to “other diseases” among men and women in the first quintile *vis a vis* those in the tenth decile. Amongst women the gain in life years due to cardiovascular diseases has contributed to reduce socioeconomic inequalities, whereas the opposite has been observed amongst men. The increase in life expectancy due to cancer and digestive diseases has been relatively similar in women from the first quintile and those in the the tenth decile. In contrast, deaths due to cancer and digestive diseases are in part responsible of an increase in the life expectancy inequality between men in the first quintile and those in the tenth decile.

A more detailed analysis looking into specific causes of deaths may shed light regarding the causes behind these patterns. In particular, it would be informative to understand to what extent the differences observed in terms of disease-specific mortality rates by sex and between socioeconomic groups are due to inequalities in terms of disease incidence or net survival (or cause-specific survival). It is paramount therefore to produce this kind of evidence to inform policy oriented recommendations aimed to reduce health inequalities.

This study has several limitations. As described in the methodology, the information about the distribution of education between the ages of 26–30 years was only available for some years. For years for which no information was available distribution by years of education was computed using linear interpolation based on the two closest adjacent known values. Moreover, as explained in the Appendix, there is a gap in the available data for the period between 1920 and 1940. A number of assumptions were therefore made to identify educational distributions before 1940 based on the information available until that year. This could introduce bias to the estimations, particularly if many changes in the educational policy took place during that period. However, overall we consider this to be unlikely, as the first educational policies with a significant possibility of shaping educational distributions were implemented in 1920 and 1929.^12^ This will begin to be reflected in the educational distribution of 26-year-old individuals in 1933 and 1946, respectively.

Research into socioeconomic inequalities in life expectancy and mortality relies on two kinds of sources of information, usually referred to as “linked” and “unlinked” data (51). Studies based on linked data use information on education from sources such as population surveys, censuses and administrative records, alongside vital statistics records at the individual level. In contrast, unlinked data studies obtain information on death counts by education from death certificates, whereas data related to population at risk by education is obtained from other sources, such as population surveys and censuses. Although the use of unlinked data is common in the literature [see for example (39, 45, 52, 53)], it is considered more prone to bias than studies based on linked data (3) as several studies have shown that data from death certificates provide biased information on educational distribution (54–58).

The data used in this study rely on information provided by the next of kin of the deceased person relating to the highest attained educational category and the number of years attained within that category. As mentioned in the Methods section, we choose to compare two educational groups (first quintile and tenth decile) because, over time, both ranks tend to match with educational categories. Arguably, this could reduce the impact of bias regarding the information on years of education within a given educational category. Moreover, is not possible to reduce the risk of bias relating to information on educational categories.

This study does not report on the precision of the life expectancy estimations. The characterization of the uncertainty of the estimations could be useful to understand how precise are these results and whether the differences observed between groups are statistically significant. Although methods to compute standard errors and confidence intervals of life expectancy estimations are available (59), most of the times these are not reported in studies assessing socioeconomic inequalities in life expectancy [see for example (3, 14, 15, 17, 18, 21, 33, 43, 46, 52, 53)] and its computation is beyond the scope of this article.

Despite these limitations, this investigation provides the first evidence of trends in socioeconomic inequality in life expectancy and lifespan variation in Chile. The findings of this study underscore the importance of monitoring the evolution of socioeconomic health inequalities and exploring new sources of information for monitoring such disparities.

## Data availability statement

Publicly available datasets were analyzed in this study. This data can be found here: Census data: (34). Censo de Población y Vivienda. https://www.ine.cl/estadisticas/sociales/censos-de-poblacion-y-vivienda/informacion-historica-censo-de-poblacion-y-vivienda and Mortality data: (35). Datos abiertos. https://deis.minsal.cl/#datosabiertos.

## Author contributions

NS-I: Conceptualization, Data curation, Formal analysis, Funding acquisition, Investigation, Methodology, Project administration, Resources, Software, Supervision, Validation, Visualization, Writing – original draft, Writing – review & editing.

## Footnotes

^1^Japan and South Korea are two countries where higher mortality has been documented among upper non-manual workers than among manual workers (4).

^2^Socioeconomic status was defined based on a multidimensional index that incorporates information about income, education, and home assets and quality of living accommodation.

^3^Top earners are pensioners receiving a monthly pension of more than USD 400, whereas low earners are those who receive less than USD 200.

^4^Studies in the US found an increasing in mortality rates among some groups which started around the year 2000, while evidence from a decreasing life expectancy among deprived groups in the UK occurs in the last decade.

^5^There are few deaths certificates (< 4%) that are not prepared by a medical doctor (36). This figure has decreased over time.

^6^Categories are as follows: limited use, low, medium-low, medium-high and high.

^7^There was a census in 2012, but due to several problems in its implementation a new census was carried out in 2017.

^8^Information available about the distribution of education by age varies between censuses. In most years it was possible to compute the distribution of education among those aged 26–30 years. In other cases information about individuals aged 25–29, or 25–34 was obtained. See Supplementary material for more details.

^9^It is assumed that at the age of 26, most individuals have completed their formal education.

^10^For instance, the difference between the first and second quintiles in 1920 was whether or not an individual was illiterate; in 1950 it was 1 year vs. 2 years of primary school; in 1980 it was completing primary school vs. continuing to secondary school; and in 2017 it was completing secondary school vs. continuing to further/higher education.

^11^In the example, about 35% of the population completed 13 years of education. Moreover, the proportion of people who attained 13 years of education and who belong to the first quintile is 5%. Therefore, 5/35 of the individuals who completed 13 years of education should be categorized in the first quintile.

^12^In 1920 it was established that all children must go to the school for 4 years before they are 13 years old, and in 1929 it became mandatory for children aged between 7 and 15 years old to complete 6 years of education.
