# Supplementary material for: Trends in socioeconomic inequalities in life expectancy and lifespan variation in Chile
Source: Front Public Health. 2024 Jun 27;12:1404410. doi: 10.3389/fpubh.2024.1404410 (PMC11236533; doi:10.3389/fpubh.2024.1404410)
Supplement: Supplementary file 1 [file Data_Sheet_1.PDF]

Information available about education attainment

The type of information and level of disaggregation by years of education and age varies between sources of information. The distribution by years of education for each year relies on information from censuses and a repeated cross-sectional survey (CASEN) which is representative of the Chilean population. Microdata was available for census and population surveys from 1990 onwards, whereas before the year 1990, census information was only available as aggregated data (.pdf files). The census of 1920, 1930, 1940 and 1952 contain abridged data by educational categories. The distribution by years of education for these years was extrapolated relying on information from the closest adjacent year for which disaggregated information was available. Table A.1 shows the information available by year and source of information, and the extrapolation method used (if applicable). The last column shows the age group for which the distribution by years of education was computed.

Table A.1. Information about education contained in census and population surveys

Year	*Source of information*	*Age group*	*Educational categories available in each census*	*Extrapolation method*	*Age group*
1920	Census	25 to 29 years	Number of individuals by literacy.	The proportion of individuals by educational category for categories different from no education was assumed to be equal to the distribution observed in 1940.<br>The proportion of individuals by years of education within each educational category was assumed to be equal to the distribution observed in 1960.	25 to 29 years
1930	Census	20 to 29 years	Number of individuals by literacy.	The proportion of individuals by educational category for categories different from no education was assumed to be equal to the distribution observed in 1940.<br>The proportion of individuals by years of education within each educational category was assumed to be equal to the distribution observed in 1960.	20 to 29 years

1940	Census	Disaggregated	Number of individuals by years of education for primary education. Number of individuals by educational category for other categories.	The proportion of individuals by years of education within each educational category for categories different from primary school was assumed to be equal to the distribution observed in 1960.	25 to 30 years
1952	Census	25 to 29 years	Number of individuals by educational category.	The proportion of individuals by years of education within each educational category was assumed to be equal to the distribution observed in 1960.	25 to 29 years
1960	Census	20 to 24 years and 25 or more	Number of individuals by years of education in the highest attained educational category for school education. Number of individuals by educational category for other categories.	A weighted average of the number of individuals by level of education in the two age groups available (20 to 24 years and 25 or more) was computed.<br><br>The total number of individuals in each educational categories was equally distributed within categories (e.g. the number of individuals with 7 years of graduate education equals the number of individuals with graduate education divided by 6).	Weighted average of age groups: 20 to 24 years and 25 or more
1970	Census	25 to 34 years	Number of individuals by abridged years of education in the highest attained educational category (e.g. 7 or 8 years of Graduate education).	The total number of individuals in each educational categories was equally distributed within categories (e.g. the number of individuals with 7 years of graduate education equals the number of individuals with 7-8 years of graduate education divided by 2).	25 to 34 years
1982	Census	25 to 29 years	Number of individuals by abridged years of education (e.g: 7 or 8 years of education).	The total number of individuals in each educational category was equally distributed within	25 to 29 years

				categories (e.g. the number of individuals with 7 years of education equals the number of individuals with 7-8 years of education divided by 2).	
1990	CASEN survey	Disaggregated	Number of individuals by years of education in the highest attained educational category.	NA	26 to 30 years
1992	Census	Disaggregated	Number of individuals by years of education in the highest attained educational category.	NA	26 to 30 years
1994	CASEN survey	Disaggregated	Number of individuals by years of education in the highest attained educational category.	NA	26 to 30 years
1996	CASEN survey	Disaggregated	Number of individuals by years of education in the highest attained educational category.	NA	26 to 30 years
1998	CASEN survey	Disaggregated	Number of individuals by years of education in the highest attained educational category.	NA	26 to 30 years
2000	CASEN survey	Disaggregated	Number of individuals by years of education in the highest attained educational category.	NA	26 to 30 years
2002	Census	Disaggregated	Number of individuals by years of education in the highest attained educational category.	NA	26 to 30 years
2006	CASEN survey	Disaggregated	Number of individuals by years of education in the highest attained educational category.	NA	26 to 30 years
2009	CASEN survey	Disaggregated	Number of individuals by years of education in the highest attained educational category.	NA	26 to 30 years
2011	CASEN survey	Disaggregated	Number of individuals by years of education in the highest attained educational category.	NA	26 to 30 years
2015	CASEN survey	Disaggregated	Number of individuals by years of education in the highest attained educational category.	NA	26 to 30 years

2017	Census	Disaggregated	Number of individuals by years of education in the highest attained educational category.	NA	26 to 30 years

Definition of years of education

The censuses, population surveys and deaths records contain infromation about the last attained educational category and the lasta ttained years of education within each category. Table A.2 shows the mapping from this information to the variable 'years of education'. The table incorporates also the corresponding ISCED-2011 (International Standard Classification of Education) codes for each educational category.

Table A.2 Mapping of educational categories to years of education

Category (in Spanish)	***Name in English***	***ISCED 2011 code***	***Years of education***
No formal education or illiteracy			0
Educación parvularia	Pre-primary Education	010, 020	0
Preparatoria (1st to 6th years)<br>Enseñanza Básica (1st to 6th years)	Primary education	100	1 to 6
Humanidades (1st to 2nd years)	Lower secondary education	244	7 to 8
Humanidades (3rd to 6th years)	a) Genereal upper secondary education.	344	9 to 12
Educación básica (7th and 8th years)	Lower secondary education	244	7 to 8
Educación media: a) humanista-científico, b) técnico profesional o c) artística	a) General upper secondary education, b) Technical upper secondary education, and c) Artistic upper secondary education.	344 and 354	9 to 12
Comercial, Industrial, Agricola, Técnico femenina, Normalista	Technical upper secondary education	354	9 to 12
Educación técnica de nivel superior	Higher technical education	554	13 to 15
Bachillerato, Licenciatura y Carrera profesional	Bachelors and Professional title	645, 646, 647, 657, 747, 748, 757, 844	13 to 19
Magíster o Doctorado	Master or Doctorate	747, 748, 844	18 to 20

Groups of diseases used in the analysis

Death records from 1991 categorises the main causes of death using ICD-9 (International classification of diseases) codes, whereas databases of 2002 and 2017 codifies causes of death according to ICD-10 codes. Table A.3 shows the codes used to categorise the groups of diseases used in the analysis.

Table A.3. Definition of groups of diseases

Group of diseases	***ICD-9***	***ICD-10***
Cancer	140-239,273.1,289.8	C00-D48
Cardiovascular	390-459 (except 427.5, 435, 446, 459)	I00-I99
Digestive	520-579	K00-K93
Infectious diseases	001-139,279.5,795.8	A00-B99,R75
Mental and behavioural	290-329	F00-F99
Respiratory	460-519, 786.0	J00-J99
Other	All but the above	All but the above

Definition of educational categories

Tables A.4 and A.5 show the distribution by years of education for different years. Cells in light yellow (green) indetify the years of education that fit into the first quintile (tenth decile). Cells in dark yellow (green) show the years of education that fit into two adjacent ranks.

Table A. 4. Distribution of education among men age 26-30 years old, by year

	1920	1930	1940	1950	1960	1970	1980	1990	2000	2010	2017
0	0.29	0.19	0.25	0.16	0.14	0.06	0.02	0.02	0.02	0.02	0.00
1	0.31	0.21	0.28	0.18	0.16	0.09	0.03	0.02	0.03	0.02	0.00
2	0.37	0.28	0.38	0.26	0.22	0.17	0.06	0.03	0.03	0.02	0.00
3	0.47	0.39	0.52	0.38	0.34	0.25	0.09	0.06	0.04	0.03	0.01
4	0.58	0.51	0.64	0.50	0.45	0.33	0.15	0.08	0.05	0.03	0.01
5	0.64	0.59	0.70	0.58	0.52	0.46	0.22	0.11	0.07	0.04	0.02
6	0.81	0.78	0.79	0.76	0.70	0.60	0.28	0.17	0.11	0.05	0.03
7	0.84	0.81	0.82	0.80	0.75	0.63	0.36	0.21	0.13	0.06	0.03
8	0.87	0.84	0.86	0.84	0.80	0.67	0.45	0.34	0.24	0.13	0.09
9	0.89	0.88	0.89	0.87	0.84	0.72	0.54	0.40	0.31	0.15	0.11
10	0.92	0.91	0.91	0.90	0.89	0.76	0.64	0.48	0.37	0.20	0.16
11	0.94	0.93	0.93	0.93	0.92	0.86	0.75	0.54	0.41	0.23	0.18
12	0.97	0.97	0.97	0.97	0.97	0.93	0.87	0.81	0.69	0.61	0.55
13	0.98	0.97	0.97	0.97	0.97	0.94	0.89	0.83	0.77	0.64	0.58
14	0.98	0.98	0.98	0.98	0.98	0.95	0.91	0.87	0.81	0.70	0.65
15	0.99	0.98	0.98	0.98	0.98	0.96	0.93	0.91	0.89	0.78	0.72
16	0.99	0.99	0.99	0.99	0.99	0.97	0.96	0.93	0.92	0.84	0.80
17	1.00	0.99	0.99	0.99	0.99	0.98	0.97	0.97	0.97	0.95	0.92
18	1.00	1.00	1.00	1.00	1.00	0.99	0.98	0.99	0.99	0.98	0.96
19	1.00	1.00	1.00	1.00	1.00	1.00	0.99	1.00	1.00	0.98	1.00
20	1.00	1.00	1.00	1.00	1.00	1.00	1.00	1.00	1.00	1.00	1.00

Table A.5. Distribution of education among women age 26-30 years old, by year

	1920	1930	1940	1950	1960	1970	1980	1990	2000	2010	2017
0	0.34	0.22	0.29	0.17	0.19	0.02	0.03	0.01	0.01	0.01	0.00
1	0.36	0.24	0.31	0.19	0.21	0.03	0.04	0.02	0.02	0.01	0.00
2	0.41	0.30	0.41	0.26	0.28	0.06	0.08	0.03	0.03	0.02	0.00
3	0.51	0.42	0.54	0.38	0.39	0.09	0.12	0.04	0.03	0.02	0.00
4	0.61	0.54	0.66	0.50	0.51	0.15	0.19	0.08	0.04	0.02	0.01
5	0.67	0.61	0.72	0.57	0.57	0.22	0.27	0.12	0.06	0.03	0.01
6	0.82	0.79	0.81	0.77	0.75	0.28	0.35	0.19	0.10	0.04	0.02
7	0.85	0.83	0.84	0.80	0.78	0.36	0.42	0.24	0.13	0.06	0.03
8	0.88	0.86	0.87	0.84	0.82	0.45	0.49	0.37	0.22	0.12	0.06
9	0.91	0.89	0.90	0.88	0.87	0.54	0.57	0.43	0.29	0.14	0.08
10	0.93	0.92	0.93	0.91	0.92	0.64	0.64	0.52	0.37	0.19	0.12
11	0.95	0.94	0.95	0.94	0.94	0.75	0.77	0.58	0.41	0.21	0.14
12	0.98	0.98	0.98	0.98	0.98	0.87	0.89	0.85	0.68	0.61	0.49
13	0.99	0.98	0.99	0.99	0.99	0.89	0.91	0.87	0.77	0.63	0.51
14	0.99	0.99	0.99	0.99	0.99	0.91	0.93	0.90	0.82	0.69	0.59
15	0.99	0.99	0.99	0.99	0.99	0.93	0.95	0.94	0.90	0.77	0.68
16	0.99	0.99	0.99	0.99	1.00	0.96	0.97	0.96	0.92	0.82	0.76
17	1.00	1.00	1.00	1.00	1.00	0.97	0.98	0.99	0.98	0.95	0.93
18	1.00	1.00	1.00	1.00	1.00	0.98	0.99	0.99	0.99	0.97	0.96
19	1.00	1.00	1.00	1.00	1.00	0.99	0.99	1.00	1.00	0.98	1.00
20	1.00	1.00	1.00	1.00	1.00	1.00	1.00	1.00	1.00	1.00	1.00
